# Efficacy of Various Facial Protective Equipment for Infection Control in a Healthcare Setting

**DOI:** 10.5811/westjem.2021.3.50516

**Published:** 2021-08-17

**Authors:** Jessica Dinsmore, Susan Brands, Steven Perry, Michael Lopez, Yutong Dong, Daniel Palasz, Jennifer Tucker

**Affiliations:** Medical College of Georgia at Augusta University, Department of Emergency Medicine, Augusta, Georgia

## Abstract

**Introduction:**

The coronavirus 2019 (COVID-19) pandemic has reinforced the importance of facial protection against droplet transmission of diseases. Healthcare workers wear personal protection equipment (PPE), including face shields and masks. Plastic face shields may have advantages over regular medical masks. Although many designs of face shields exist, there is a paucity of evidence regarding the efficacy of shield designs against droplet transmissions. There is even less published evidence comparing various face shields. Due to the urgency of the pandemic and the health and safety of healthcare workers, we aimed to study the efficacy of various face shields against droplet transmission.

**Methods:**

We simulated droplet transmission via coughing using a heavy-duty chemical spray bottle filled with fluorescein. A standard-adult sized mannequin head was used. The mannequin head wore various face shields and was positioned to face the spray bottle at either a 0°, 45°, or 90° angle. The spray bottle was positioned at and sprayed from 30 centimeters (cm), 60 cm, or 90 cm away from the head. These steps were repeated for all face shields used. Control was a mannequin that wore no PPE. A basic mask was also tested. We collected data for particle count, total area of particle distribution, average particle size, and percentage area covered by particles. We analyzed percent covered by particles using a repeated measures mixed-model regression with Tukey-Kramer pairwise comparison.

**Results:**

We used least square means to estimate the percentage area covered by particles. Wearing PPE regardless of the design reduced particle transmission to the mannequin compared to the control. The LCG mask had the lowest square means of 0.06 of all face-shield designs analyzed. Tukey-Kramer pairwise comparison showed that all PPEs had a decrease in particle contamination compared to the control. LCG shield was found to have the least contamination compared to all other masks (P < 0.05).

**Conclusion:**

Results suggest the importance of wearing a protective covering against droplet transmission. The LCG shield was found to decrease facial contamination by droplets the most of any tested protective equipment.

## INTRODUCTION

Based on current evidence, coronavirus disease 2019 (COVID-19) is transmitted between people through close contact and respiratory droplets.^1^ Airborne transmission occurs through coughing, sneezing, or talking with infected droplets landing on a mucosal surface or being inhaled into the lungs via nasal or oral passage.^2^ For the lay person, precautions such as maintaining a minimum of six feet distance from others, performing hand hygiene, and wearing a medical mask have been recommended.^3^ However, in healthcare settings providers frequently perform aerosolizing procedures (ie, tracheal intubation, non-invasive ventilation, bronchoscopy, etc) and provide clinical care requiring close physical contact. Because of additional risk factors for transmitting and contracting the disease in healthcare settings, the World Health Organization (WHO) has specific guidelines in place to prevent or limit COVID-19 transmission in these settings.

The WHO guidelines include the following: early recognition and isolation of suspected and confirmed COVID-19 cases; applying standard precautions for all patients entering the facility; and applying empiric additional precautions for suspected and confirmed cases of COVID-19.^4^ The standard precautions are in place to reduce transmission from both recognized and unrecognized sources and should be used in the care of all patients: diligent hand washing; maintaining greater than six feet of distance if possible, etc. Additional precautions that are required if a patient is either a suspected or confirmed case include contact and droplet precautions, as well as airborne precautions in aerosol-generating procedures.^5^ Although these precautions vary by hospital, contact precautions most commonly include a gown and gloves; droplet precautions include a gown, gloves, standard mask, and eye protection; and airborne precautions include all those of droplet in addition to donning of a fit-tested N-95 or higher-level respirator prior to room entry. Specific to the novel coronavirus, the US Centers for Disease Control and the Occupational Safety and Health Administration have recommended that healthcare workers use full-face shields to protect against exposure to COVID-19.^6^,^7^ This recommendation is secondary to their covering of the three major areas of transmissibility: the eyes, nose, and mouth.

Given the critical areas of transmissibility they are protecting, it is important to understand how effective face shields act as physical barriers in limiting the spread of infectious particles. Although not always appearing complex, face shields are subject to strict regulation. The ANSI/ISEA Z.87.1-2015 standard in the US specifies physical features of a face shield that must maintain proper visual power, resistance to high-velocity impacts, and protection from droplets and splashes.^8^ However, given the national shortage of personal protection equipment (PPE) that developed during the pandemic, on March 2, 2020, the US Food and Drug Administration (FDA) granted an emergency use authorization (EUA) for personal respiratory protective devices during the COVID-19 outbreak. These EUAs are typically put in place in “disaster” situations, or when environmental demand outpaces medical response, or during public health emergencies with significant potential to affect the health and security of US citizens. On February 4, 2020, the Secretary of the Department of Health and Human Services (HHS) determined that the rapid increase in the spread of the novel coronavirus fit such a definition.^9^

**Population Health Research Capsule**

What do we already know about this issue?

*Face shields are effective pieces of health worker protective equipment and reduce exposure to droplet-borne pathogens, but relative efficacy is difficult to assess*.

What was the research question?

*What is the efficacy of a variety of face shields in reducing airborne droplet exposure for the wearer?*

What was the major finding of the study?

*We found that the LCG shield was the most protective of the face shields tested*.

How does this improve population health?

*We reinforce the protective value of simple, low-cost PPE, illustrate trends in efficacy between various designs, and develop a low-cost, reproducible testing method*.

As a result, a variety of alternatives to traditional FDA-cleared masks became available over the course of 2020, many of which were difficult to manufacture, financially unreasonable, or potentially less effective in preventing transmission of respiratory droplets to the wearer. The typical face shield design includes a flat plastic shield, a headband, and brow foam. Most are designed to be low cost, discarded after a single use, and mass-producible.^10^ Some, such as the Prusa shield, are intended for multiple use and considered superior in fit and function compared to disposable shields. However, as demand for face shields continued to increase and PPE shortages proliferated across the country, lower-quality face shields were less likely to be discarded after a single use, raising concern as to whether these lower quality shields maintained their efficacy and further increasing demand for more reliable, multi-use shields.

While the benefit of a face shield as a whole has been confirmed through large review studies as a useful physical barrier in limiting the spread of infectious particles, such as in Roberge’s 2016 study, Roberge also found that fit, length, and type of face shield made a significant difference in barrier effectiveness.^10^ Other variables such as aerosolized particle size, distance from simulated cough, and air-time of aerosolized particles play a major role in determining effectiveness of face shields. Despite knowing that many factors contribute to an effective face shield, previous studies comparing shield efficacy have lacked a standard test or measure of face shield effectiveness. Therefore, the purpose of this study was to evaluate the efficacy of various face shields used in the healthcare setting for infection control at preventing droplet dispersal and contamination of the end user, as well as other PPE worn concomitantly with the face shields.

## METHODS

We used a cough simulation model to evaluate the efficacy of various facial PPE. This simulation involved a heavy-duty chemical spray bottle filled with fluorescein to simulate the respiratory droplet dispersal of a cough as well as an anatomically correct, adult-mannequin head outfitted with each of the facial PPE devices that were being tested. The fluorescein dye allowed the droplets that landed on the mannequin’s face to be visualized and photographed under fluorescent light. We then analyzed these photographs to determine the percentage area of the mannequin head that the droplets covered.

The spray bottle was positioned so that the nozzle was at the same height as the brow of the mannequin. This spray bottle stand was then positioned 30 centimeters (cm), 60 cm, or 90 cm away from the head. The head was positioned to face the spray bottle at a 0°, 45°, or 90° angle. This set-up was then used to perform five spray tests for each of the nine different angle and distance combinations. After each spray, the PPE was carefully removed from the mannequin’s head, and the resulting fluorescent droplet pattern was photographed from both a front facing and left-side facing view.

The images were cropped in such a way that the edges of the mannequin’s face were equidistant from the edges of the picture’s frame in each analyzed photograph. We then used Image J, an open-source, Java-based image processing software developed at the National Institutes of Health, to calculate percentage area of the cropped face covered by fluorescent particles. All front-facing images were identical in area to allow for facial surface area covered in fluorescein to be accurately compared. The same was true for the side-facing images. We ran the images through Image J’s color split function to ensure that only the green fluorescent particles would be read and analyzed by the program. We then applied the Otsu auto threshold function along with the B&W (black and white) setting to the images prior to using the “analyze particle” functions. The “analyze particle” function was then run with a threshold size of 0-infinity pixels squared and a circularity of 0.00–1.00. The results of this analysis included particle count, total area of particle distribution, average particle size, and percentage area covered by particles. This data output was then saved and compiled for each set of data.

The statistical analyses were two-tailed and conducted at a significant level of 0.05 using SAS 9.4 (SAS Institute, Cary, NC). We used Tukey-Kramer pairwise multiple comparisons in repeated measures analysis based on mixed model regression to compare the “percentage area covered by particles” between a mask and the control and between the masks. These comparisons were performed on the average of the percentage area covered by the particles across the five spray tests that were performed on each control and mask set-up. We measured the average particle size of the droplets by including a ruler in the photograph of a 0° angle, front-facing control at 30 cm, 60 cm, and 90 cm. The set scale function within Image J was then used to convert micrometers (μm) to pixels. The images were analyzed as above, but the distribution function was run after the particle analysis function. This generated the mode and range of the particle size for the droplets in micrometers squared.

## RESULTS

The average percentage area covered by particles for each mask, angle, and spray distance are shown in Table 1 (front view) and Table 2 (side view). As the “percentage area covered by particles” represents the amount of contamination by respiratory droplets, this value will be simplified to particle contamination for the remainder of the paper. The model estimated least square means (LS means) for each facial PPE and the control is depicted in Table 3. These results demonstrate that all of the facial PPE had less particle contamination than the control. Of the facial PPE tested, the LCG mask (LCG Industries Ltd., Faridabad, Haryana, India) had the lowest amount of particle contamination with a LS mean of 0.06.

Table 4 depicts the results of the Tukey-Kramer multiple pairwise comparison for the percentage area covered by particles. These results show that all the facial PPE had a statistically significant decrease in particle contamination when compared to the control. This table also depicts the relative efficacy of the facial PPE when compared against all the masks tested in this study. The facial PPE that had a shield – surgical mask, Medline, PRUSA, and LCG – were also shown to offer significantly more protection compared to the basic mask. When the PPE with shields were compared against each other, we found no statistically significant difference in the protection offered between the surgical mask, the PRUSA face shield (which can be manufactured with a three-dimensional printer [Prusa Research, Czechoslovakia]), and the Medline face shield (Medline Industries, Inc., Northfield, IL). The LCG shield, however, was shown to be statistically more protective than all other forms of facial PPE tested in this study.

**Figure 1:**
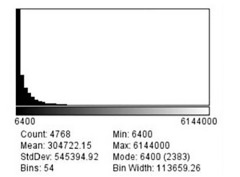
Particle distribution sizes for 30 centimeters spray in micrometers squared. *StdDev*, standard deviation; *min*, minimum.

**Figure 2:**
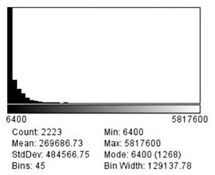
Particle distribution sizes for 60 centimeters spray in micrometers squared. *StdDev*, standard deviation; *min*, minimum.

**Figure 3:**
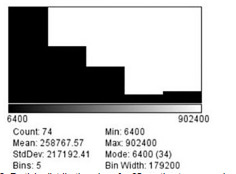
Particle distribution sizes for 90 centimeters spray in micrometers squared. *StdDev*, standard deviation; *min*, minimum.

The set scale function determined that there were 0.0125 pixels per μm, which can also be converted to 80 μm per pixel. As none of the particles on the mannequin face visually measured more than 2 millimeters (mm) in diameter, a limit of 6,250,000 μm squared (μm^2^) was set as the maximum size for the distribution function when analyzing these images for a second time. This limit excludes any particle greater than 2.5 mm in diameter and was imposed to exclude particles that the program mis-read as being one large particle with tiny gaps, rather than individual particles. Figures 1, 2, and 3 demonstrate that the smallest particle area is 6400 μm^2^, or 80 μm in diameter. This number also happens to be the mode for the particle area sizes across all three distances.

## DISCUSSION

There is poor evidence and regulatory specificity as to the appropriate size, design, or performance standards for face shields for healthcare workers and others potentially exposed to contaminating respiratory and aerosol droplets. Previous studies have described the difficulty in accurately simulating the human cough and associated droplet size distribution, requiring complex equipment. Our study specifically employed a low-cost method to compare the efficacy of multiple. protective facial barriers by quantifying the volume of droplets reaching the face. Xie et al demonstrated that 63% of the particles in a cough were between 50–150 μm in diameter with 64% of the particles being less than 100 μm in diameter overall. After trial of multiple devices to simulate a cough, we found that a spray bottle designed for use in automotive detailing, the ACC_130 Professional (Chemical Guys, Gardena, CA) approximated a distribution of droplet sizes in a human cough.

All face shields showed a statistically significant reduction in facial droplet coverage vs no mask. When shields were compared against one another, we found no significant difference between the protection offered by the surgical mask, the PRUSA face shield, and the Medline face shield. The LCG shield, however, was shown to be statistically more protective than all other forms of facial PPE tested in this study. The order of mask efficacy based on the smallest to greatest area of face covered by fluorescein droplets is as follows: the LCG shield (most effective); Medline face shield; surgical mask with attached eye shield; Prusa face shield; and basic surgical mask (least effective). Of note, the LCG shield extends significantly inferiorly beyond the chin and wraps posteriorly past the temples.

Our literature review found no studies to similarly demonstrate an easily employable, low-cost method for comparing the efficacy of facial barriers and shields designed to protect the wearer. This approach may allow individuals and institutions to better select the PPE they acquire for their workers. Similar techniques may help to refine regulatory guidance regarding specifications and standards for such protective equipment to improve workplace safety for healthcare providers. Since the beginning of the COVID-19 pandemic and the healthcare system’s response, institutions and providers have encountered countless types of PPE with significant confusion about their relative efficacy or durability. Given the variation in design, quality, and efficacy observed within a limited set of face shields, we encourage employment of this technique by future researchers to better define the ideal design for face shields to protect against communicable diseases.

## LIMITATIONS

Limitations of this study include limited statistical power, imperfect cough simulation, and difficulty detecting the smallest aerosol particles. Statistical power was limited by a limited number of trials performed on each mask from each position. Regarding droplet size detection, the Image J program cannot measure particles that are smaller than a pixel. As each pixel was 80 μm in diameter or 6400 μm^2^, the microscopic particles were often measured as one larger particle, especially when these smallest particles were clustered close together within the image. However, this is not expected to significantly affect surface area covered by particles between the different masks as the analysis of particles was done identically for every image.

Previous studies have also identified another discrepancy with these smaller particles in that they are more likely to circulate in the air around face shields for a longer period of time than direct-trajectory, larger particles. Hence, the smallest particles can continue to settle on mucosa minutes after they are expelled. While we allowed an equal pause after each spray to allow for smaller particles to settle prior to stepping into the study zone and removing the PPE to capture our images, allowing adequate time for the settling of these smaller particles as well as the accurate recording of them is another limitation of this study. Regardless, we believe this limitation could be mitigated but not eliminated by using higher resolution cameras, taking multiple pictures of facial sub-areas at different focal lengths and zoom distances, allocating multiple minutes for particle settlement, and more numerous and distributed ultraviolet lighting sources to increase droplet fluorescence intensity.

## CONCLUSION

The COVID-19 pandemic has emphasized the need to find a standardized method for measuring face shield effectiveness. It is challenging to simulate viral particle spread due to the many variables involved in the spread and visualization of microscopic particles. In this pilot study, we designed a simulated “head” and “cough,” along with a standard method of particle exposure and analysis, to quantify how well face shields can prevent the spread of aerosolized particles potentially carrying infectious contagions. We then compared the effectiveness of face shields with different sizes, shapes, and fits. Our method of analysis differentiated face shields quantitatively. We found that the LCG face shield was the most effective in reducing particle exposure because of its peripheral covering. The methods used here may also be useful in comparing other forms of personal protective equipment. This is critically important in its relevance not only to protection of high-risk persons during the COVID-19 pandemic, but also to the day-to-day safety of high-risk persons in all infectious disease settings.

## Supplementary Information



## Figures and Tables

**Table 1:** Front view. Average percentage of facial area covered by fluorescein particles for mask type and spray distance.

Mask type	Degree	Percent area 30cm	Percent area 60cm	Percent area 90cm
Control	0	21.23	9.45	0.29
	45	12.49	3.55	0.087
	90	4.02	0.70	0.014
Basic	0	7.08	0.12	0.0038
	45	5.11	1.31	0.016
	90	2.89	0.58	0.021
Surgical	0	0.011	0.0094	0.0029
	45	1.59	0.70	0.0060
	90	2.39	0.32	0.0027
Medline	0	0.0029	0.00034	0.0019
	45	0.30	0.0023	0.00020
	90	1.81	0.81	0.0048
Prusa	0	0.023	0.012	0.003
	45	1.29	0.36	0.0069
	90	2.86	0.78	0.016
LCG	0	0.012	0.0021	0.00060
	45	0.0095	0.0019	0.000086
	90	0.0098	0.028	0.0011

*cm*, centimeter.

**Table 2:** Side view. Average percentage of facial area covered by fluorescein particles for mask type and spray distance.

Mask type	Degree	Percent area 30cm	Percent area 60cm	Percent area 90cm
Control	0	4.29	2.06	0.0070
	45	15.64	4.92	0.078
	90	16.63	5.67	0.11
Basic	0	0.97	0.0013	0.0030
	45	9.16	2.00	0.11
	90	14.49	5.16	0.205
Surgical	0	0.00064	0.00080	0.00039
	45	4.45	1.27	0.10
	90	12.28	3.90	0.097
Medline	0	0.00047	0.00048	0.00036
	45	0.69	0.012	0.0030
	90	11.64	5.70	0.098
Prusa	0	0.00056	0.00062	0.0013
	45	3.25	1.08	0.038
	90	14.89	4.79	0.098
LCG	0	0.00065	0.00039	0.000068
	45	0.019	0.0040	0.00072
	90	0.16	0.90	0.0074

*cm*, centimeter.

**Table 3:** Least square (LS) means for each facial mask.

Group	LS means	Standard error
Basic mask	2.74	0.75
Control	5.62	0.75
LGG shield	0.06	0.75
Medline	1.17	0.75
Prusa	1.64	0.75
Surgical mask	1.51	0.75

**Table 4:** Tukey-Kramer multiple pairwise comparison for percentage area covered of particles.

Group	T-value	Adjusted *P*-value
Basic mask		
Control	−7.54	0.0000**
LGG shield	6.98	0.0000**
Medline	4.08	0.0007**
Prusa	2.87	0.0493**
Surgical mask	3.21	0.0177**
Control		
LGG shield	14.52	0.0000**
Medline	11.63	0.0000**
Prusa	10.41	0.0000**
Surgical mask	10.75	0.0000**
LGG shield		
Medline	−2.89	0.0459**
Prusa	−4.11	0.0007**
Surgical mask	−3.77	0.0025**
Medline		
Prusa	−1.22	0.8273
Surgical mask	−0.88	0.9518
Prusa		
Surgical mask	0.34	0.9994

** Significant value, *p*<0.05
