# Supplementary material for: Efficacy of Various Facial Protective Equipment for Infection Control in a Healthcare Setting
Source: West J Emerg Med. 2021 Aug 17;22(5):1045–50. doi: 10.5811/westjem.2021.3.50516 (PMC8463064; doi:10.5811/westjem.2021.3.50516)

Index

Diagrams of Spray Test Set-up………………………………….2

Images and Descriptions of Masks and Face Shields….….…….4

Tables of Area of Covered by Particle for Each Spray Test…….7

Control……………..7

Basic Mask…….…..8

Surgical Mask…….10

Medline…………...11

Prusa………………13

LCG………………14

Diagrams of Spray Test Set-up:


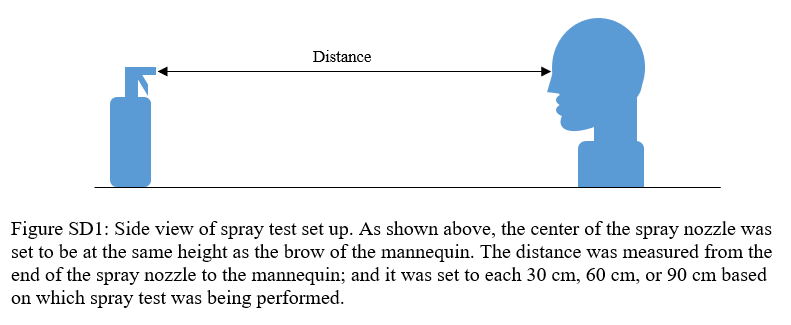


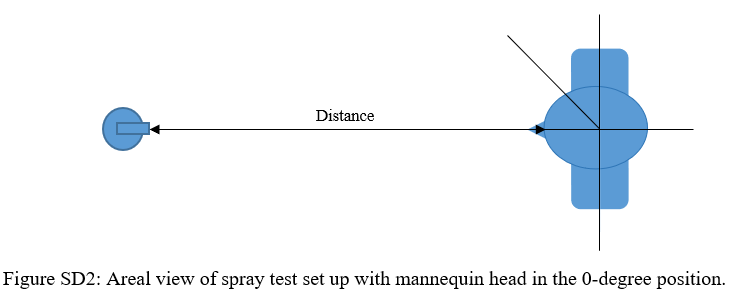


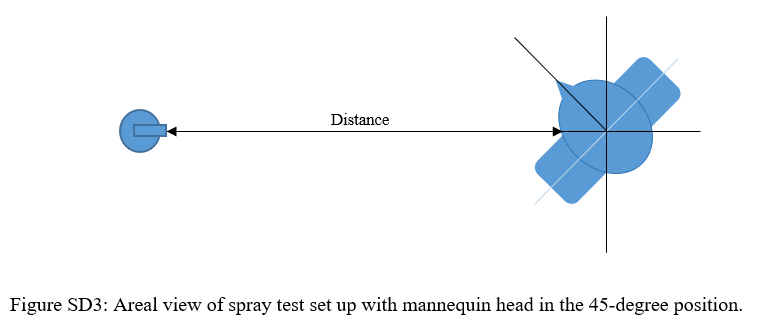


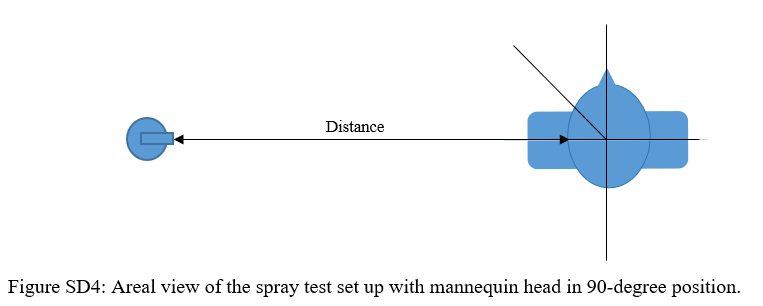


Images and Descriptions of Masks and Face Shields:


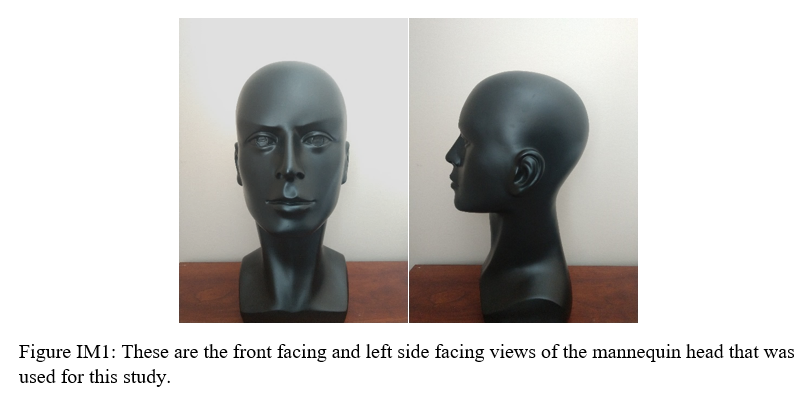


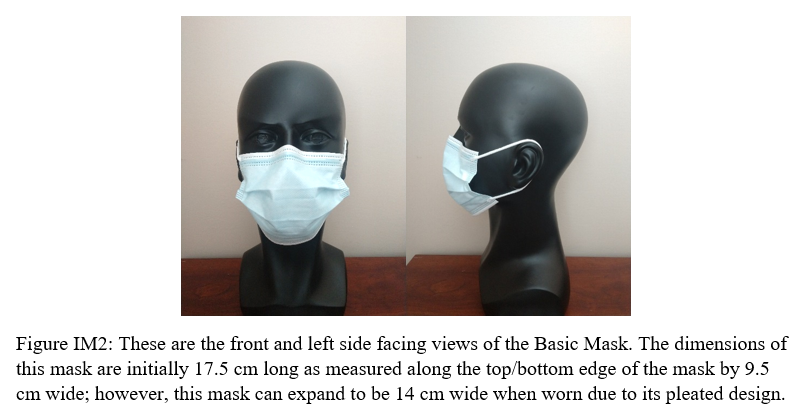


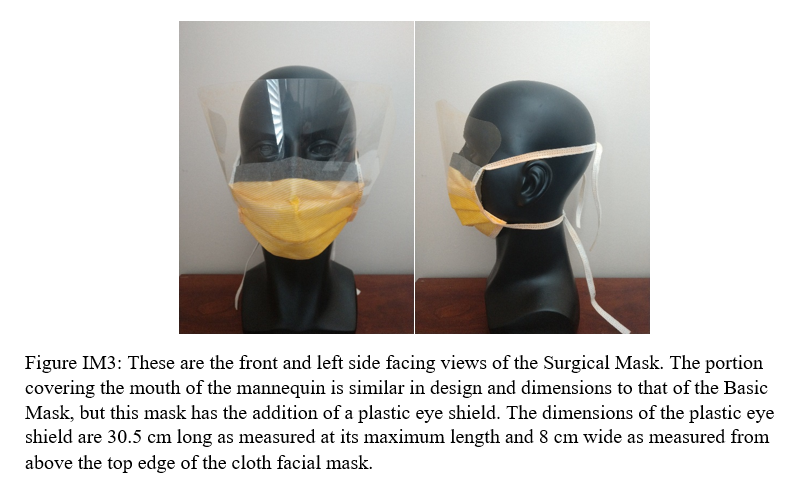


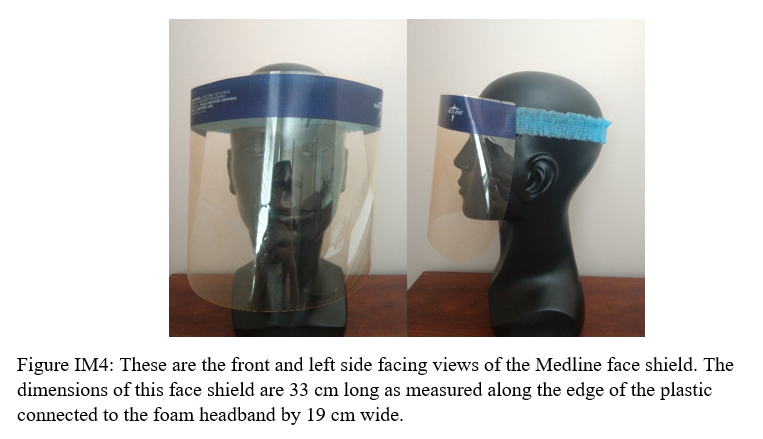


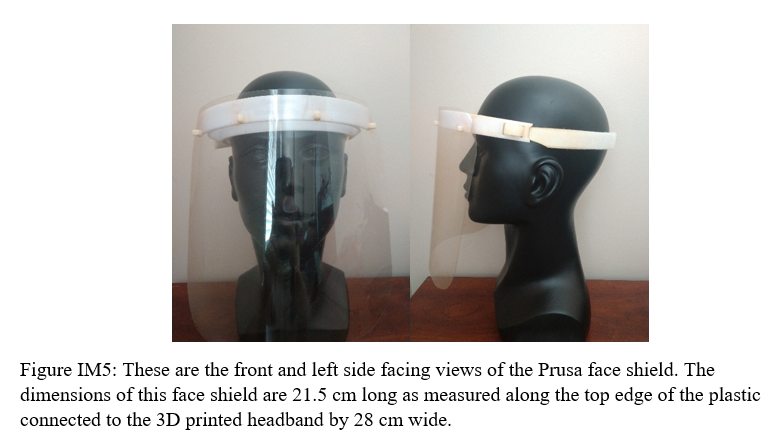


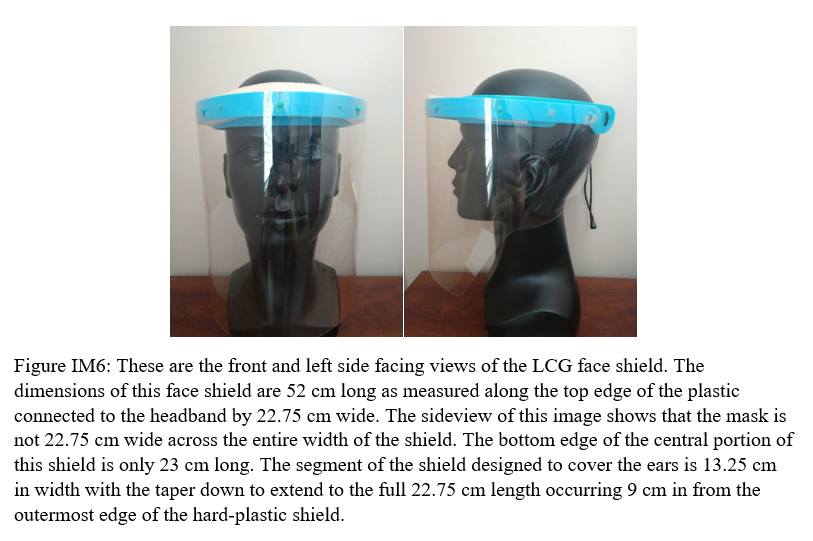


Tables of Area of Covered by Particle for Each Spray Test:

Control:


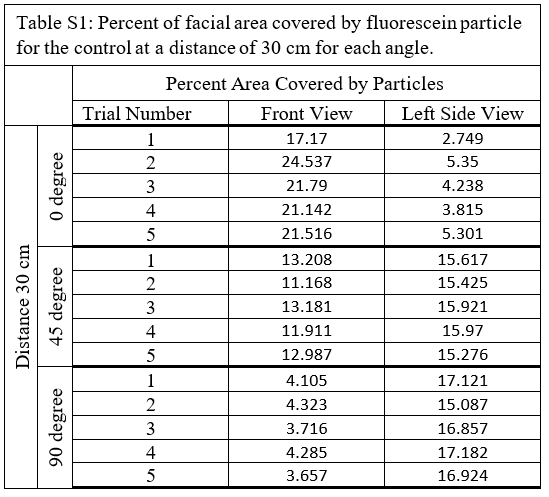


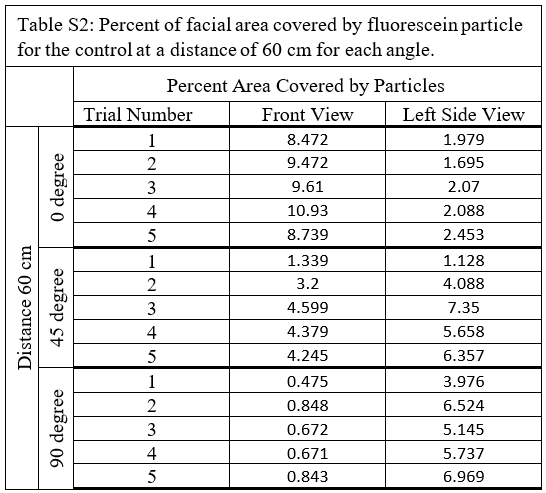


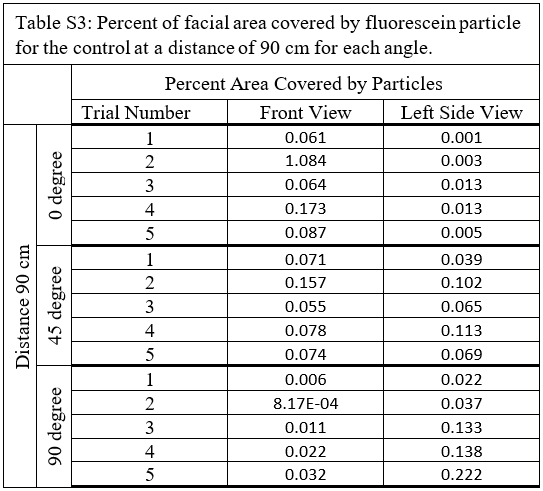


Basic Masks:


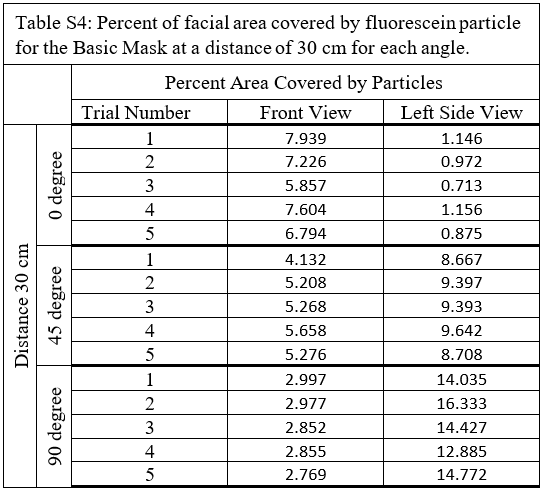


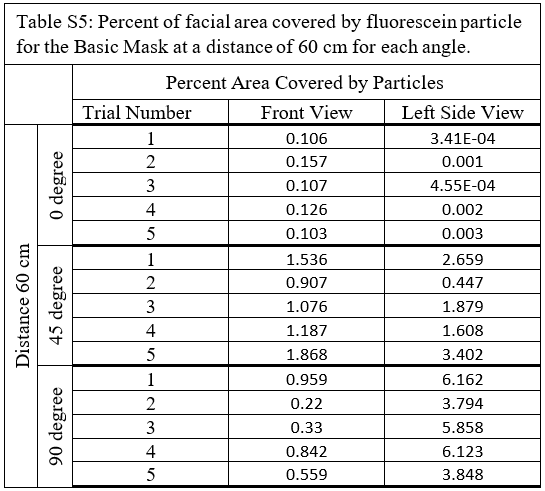


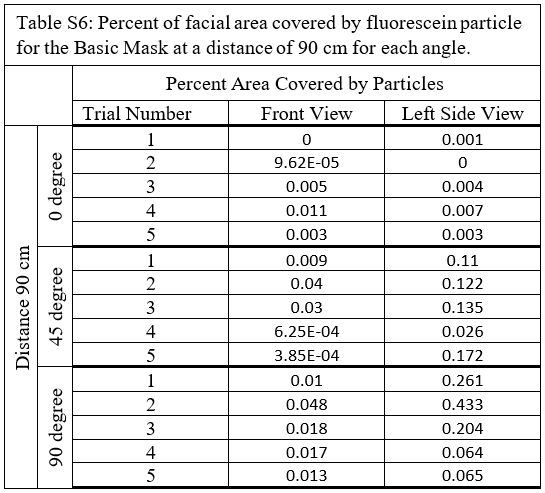


Surgical Mask:


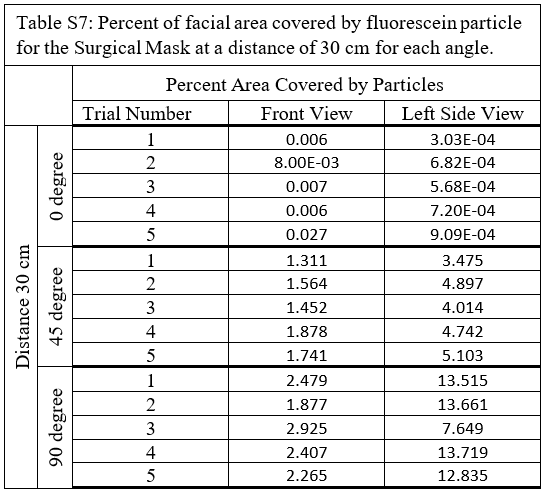


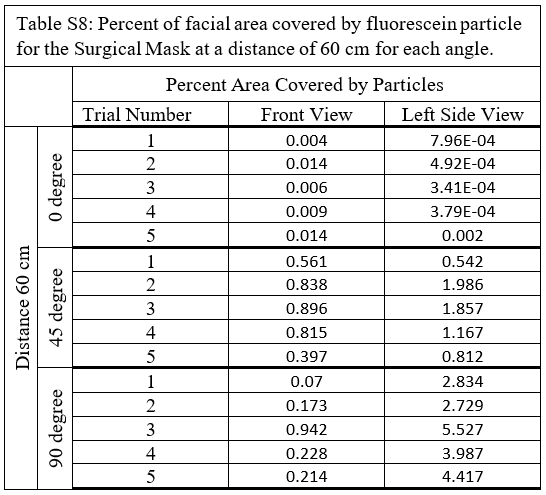


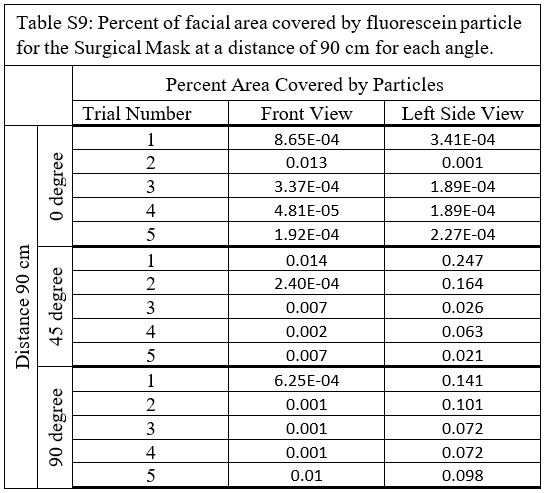


Medline face shield:


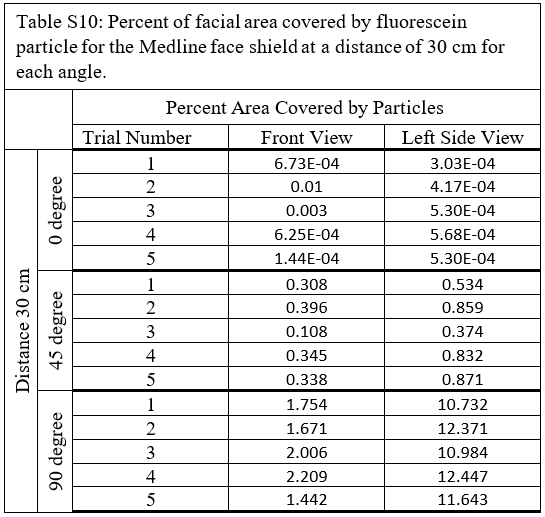


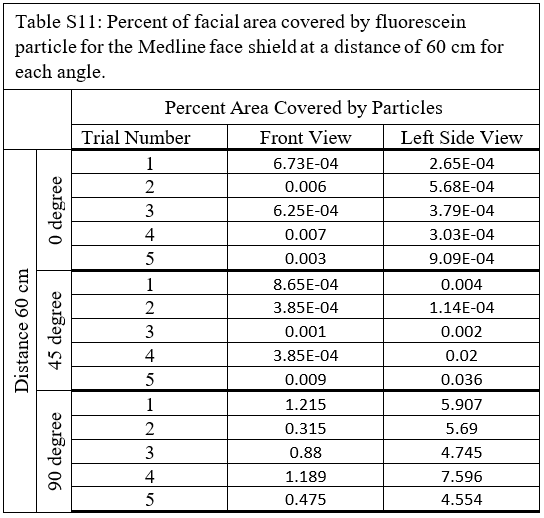


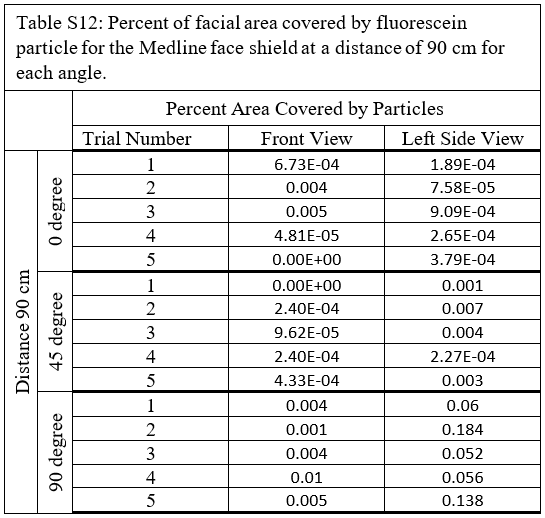


Prusa face shield:


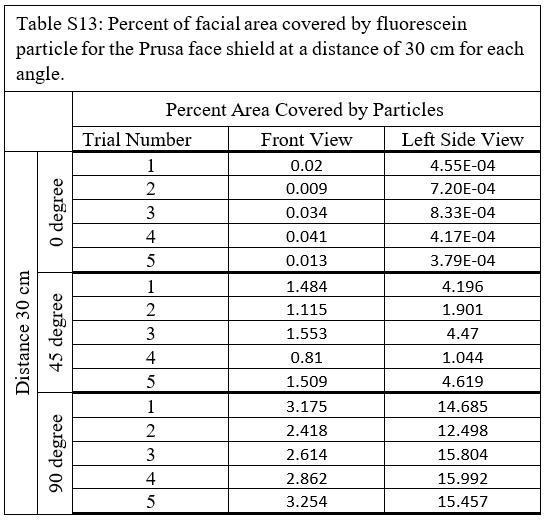


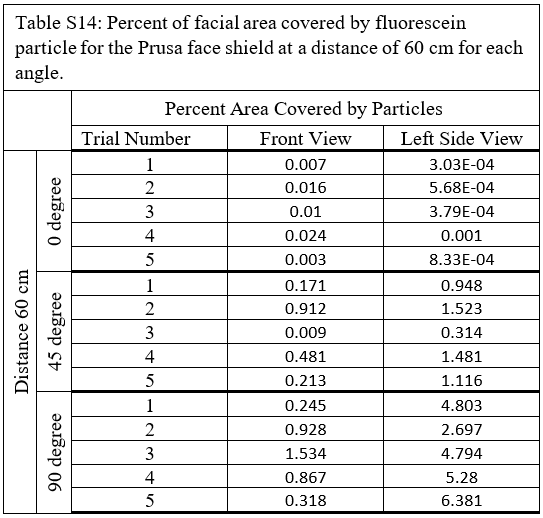


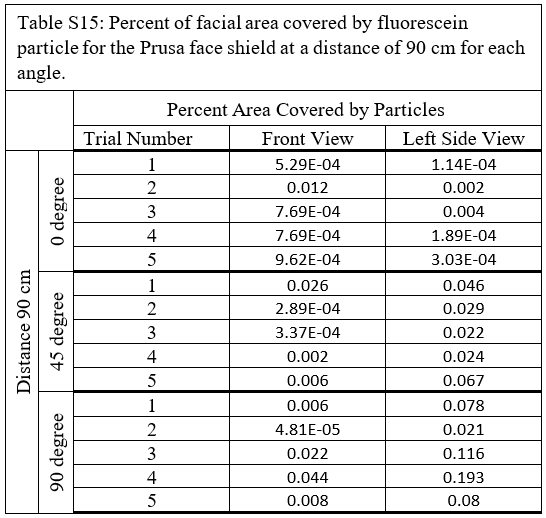


LCG face shield:


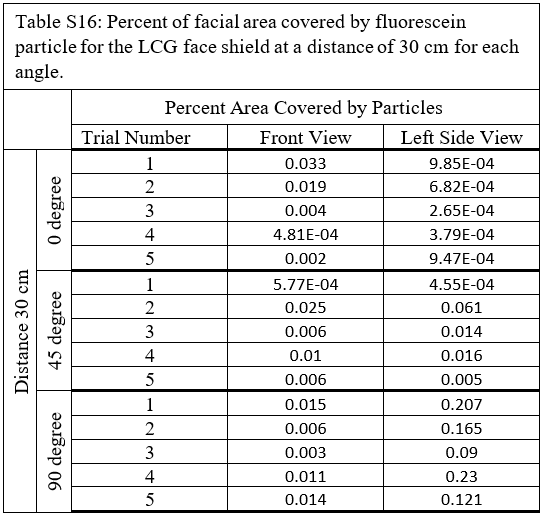


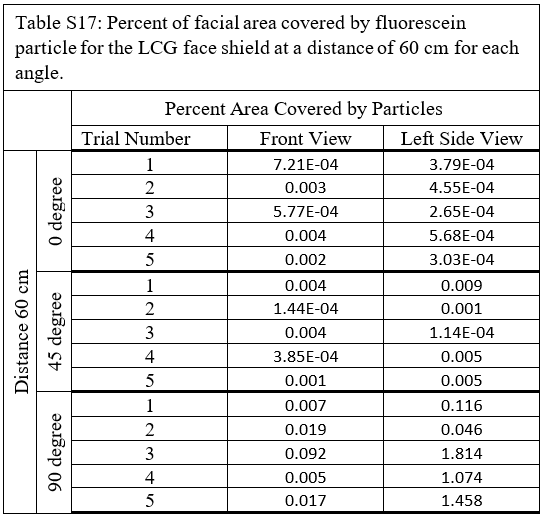


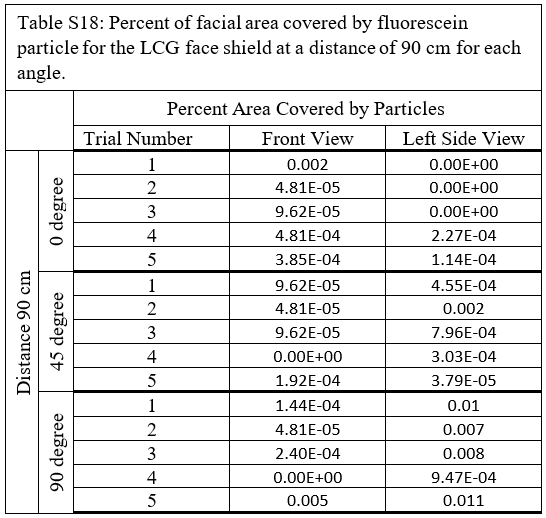

Supplement: Supplementary file 1 [file wjem-22-1045-s001.docx]
